# Spatial heterogeneity of extensively drug resistant-tuberculosis in Western Cape Province, South Africa

**DOI:** 10.1038/s41598-022-14581-4

**Published:** 2022-06-27

**Authors:** Karla Therese L. Sy, Sarah V. Leavitt, Margaretha de Vos, Tania Dolby, Jacob Bor, C. Robert Horsburgh, Robin M. Warren, Elizabeth M. Streicher, Helen E. Jenkins, Karen R. Jacobson

**Affiliations:** 1 Department of Epidemiology, Boston University School of Public Health, Boston, MA USA; 2 Department of Biostatistics, Boston University School of Public Health, Boston, MA USA; 3 DSI-NRF Centre of Excellence for Biomedical Tuberculosis Research/South African Medical Research Council Centre for Tuberculosis Research, Division of Molecular Biology and Human Genetics, Faculty of Medicine and Health Sciences, Stellenbosch University, Cape Town, South Africa; 4 National Health Laboratory Service, Cape Town, South Africa; 5 Department of Global Health, Boston University School of Public Health, Boston, MA USA; 6 Section of Infectious Diseases, School of Medicine and Boston Medical Center, Boston University, Boston, MA USA

**Keywords:** Public health, Epidemiology

## Abstract

Tuberculosis (TB) remains a leading infectious disease killer globally. Treatment outcomes are especially poor among people with extensively drug-resistant (XDR) TB, until recently defined as rifampicin-resistant (RR) TB with resistance to an aminoglycoside (amikacin) and a fluoroquinolone (ofloxacin). We used laboratory TB test results from Western Cape province, South Africa between 2012 and 2015 to identify XDR-TB and pre-XDR-TB (RR-TB with resistance to one second-line drug) spatial hotspots. We mapped the percentage and count of individuals with RR-TB that had XDR-TB and pre-XDR-TB across the province and in Cape Town, as well as amikacin-resistant and ofloxacin-resistant TB. We found the percentage of pre-XDR-TB and the count of XDR-TB/pre-XDR-TB highly heterogeneous with geographic hotspots within RR-TB high burden areas, and found hotspots in both percentage and count of amikacin-resistant and ofloxacin-resistant TB. The spatial distribution of percentage ofloxacin-resistant TB hotspots was similar to XDR-TB hotspots, suggesting that fluoroquinolone-resistace is often the first step to additional resistance. Our work shows that interventions used to reduce XDR-TB incidence may need to be targeted within spatial locations of RR-TB, and further research is required to understand underlying drivers of XDR-TB transmission in these locations.

## Introduction

Tuberculosis (TB) remains a leading global infectious disease killer, with an estimated 1.4 million deaths in 2019^1^. Further exacerbating this global public health crisis is drug-resistant TB, which requires more complex and lengthy treatments, and has greater mortality than drug-susceptible disease. In 2018, 6.2% of people with multidrug-resistant (MDR; resistance to both rifampicin and isoniazid) TB were found to have extensively drug-resistant (XDR; MDR plus resistance to an aminoglycoside and a fluoroquinolone, the definition applicable to the time period of our data)^1^ TB.

South Africa is one of the five countries with the greatest burden of drug-resistant TB, with an estimated MDR-TB incidence of 23/100,000 individuals in 2019^1^. In South Africa, MDR and rifampicin-resistant (RR; which triggers second-line drug treatment and is often a proxy for MDR-TB) TB accounted for 3.4% of new and 7.1% of previously treated patients in 2018^1^. Despite increased political commitment and funding to provide earlier testing and treatment for drug-resistant TB, in 2018 only 57% of South Africans with RR-TB were tested for second-line drug resistance and only 58% of those identified with XDR-TB started second-line treatment^1^. MDR-TB/RR-TB has been shown to be spatially heterogenous across South Africa, including within provinces and sub-districts^2–5^, and in other countries such as Peru, Ethiopia, China, Moldova, Georgia, and Portugal^6–12^. On a population level, it is unclear whether XDR-TB is a relatively consistent proportion of all RR-TB, or whether there are geographic XDR-TB hotspots within RR-TB hotspots. The only studies of the spatial heterogeneity of XDR-TB to date have been in South Africa’s KwaZulu-Natal Province; these, though, did not explicitly adjust for underlying MDR/RR-TB burden^5,13–15^. Although rapid tools to detect rifampicin resistance have contributed to increased identification and early treatment of MDR-TB^16^, knowledge of where XDR-TB geographic hotspots (i.e. areas of higher than average burden) is needed to target additional resources and interventions, and reduce treatment delays and associated poor outcomes^1,17,18^.

In our study, we aimed to characterize the spatial heterogeneity of XDR-TB (defined as RR-TB with resistance to an aminoglycoside and a fluoroquinolone) and pre-XDR-TB (defined as RR-TB plus resistance to either an aminoglycoside or a fluoroquinolone) in the Western Cape Province, South Africa. We also calculated the number of individuals with RR-TB and the percentage of these individuals whose specimen had second-line drug susceptibility testing. We also assessed whether our findings were robust to potential biases.

## Methods

### Study setting and participants

Individuals with TB were identified from routinely collected laboratory data from the Western Cape National Health Laboratory Services (NHLS) in South Africa between January 1, 2012 to July 31, 2015. Our method of data abstraction and processing has been previously described^19,20^. The NHLS processes tests for the South African public health care system and accounts for 93% of all TB tests nationally^21^. During the study period, when a person was evaluated for TB disease, often with symptoms suggestive of TB, two clinical specimens were sent out for testing; the first sample was tested with GeneXpert MTB/RIF assay (Xpert) (Cepheid, Sunnyvale, CA, USA). If RR-TB (defined as TB with resistance to rifampicin) was detected, the second sample was used for confirmatory testing for *Mycobacterium tuberculosis* (*Mtb*) presence and first-line resistance with smear microscopy, culture (Mycobacterial Growth Indicator Tube, MGIT), or LPA (GenoType® MTBDR*plus*, Hain Lifescience). Phenotypic drug susceptibility testing (DST) was done on 7H10 media containing 2 µg/ml ofloxacin (for fluoroquinolone resistance) or 4 µg/ml Amikacin (for aminoglycoside drug resistance testing) to assess second line drug (SLD) resistance. Fluoroquinolones and injectable aminoglycosides were the two drug classes that constituted the backbone of second line therapy at the time, and these two drugs were the standard to capture phenotypic resistance in these classes. Resistance to either drug was associated with worse treatment response outcomes. In our study, we designated individuals with RR-TB as laboratory confirmed resistance to rifampicin based on Xpert, LPA, or phenotypic DST result on culture. We did not exclude RR-TB cases with no second-line DST due to the high second-line DST coverage, which was 82% (Supplementary Fig. 1). We defined XDR-TB as RR-TB with the additional resistance to both ofloxacin and amikacin. As we wanted to consider the spectrum of drug resistance, we also conducted all analyses for an XDR-TB patient group that included pre-XDR-TB, defined as RR-TB resistant to either ofloxacin or amikacin, hereafter referred to as “pre-XDR-TB/XDR-TB”. Therefore, XDR-TB is defined as resistant to at least rifampin plus ofloxacin or amikacin, a subset of RR-TB which requires only resistance to rifampin. Pre-XDR- and XDR-TB status were based on any second-line DST result, which were not always on the day of RR-TB diagnosis. Our study therefore consisted of three, nested drug-resistant TB groups: RR-TB, pre-XDR-TB/XDR-TB, and XDR-TB.

The NHLS centralized laboratory database lacks unique individual identifiers; thus, we used a person-matching algorithm to link specimens to individuals, also described elsewhere^19,20^. Since our study period was three years, we defined any multiple positive tests to be due to one disease episode. The NHLS database includes the facility code that each sample specimen was submitted from. We used these facility codes to identify a “home clinic” for each individual as a proxy for where the person was living at the time of diagnosis, which is a reasonable approximation given previous research has shown that TB patients do not travel far distances to receive their diagnosis in the Western Cape^19^, especially after decentralization of MDR-TB/RR-TB care^22^. This home clinic was defined as the location of their clinic visit closest in time to their RR-TB diagnosis. We removed individuals with samples submitted exclusively from non-clinic locations (hospital, prison, other) on the assumption that individuals from these locations originate from a broader community and do not reflect spatial distribution of residence at time of testing^4^. Using the NHLS and National Department of Health (NDoH) reference lists, we determined the facility name, type, and geo-coordinates of each clinic. Two researchers from Boston University manually validated the geo-coordinates on Google Maps, and South African researchers/healthcare providers resolved discrepancies if any occurred. Moreover, facilities that overlapped in geocoordinates and were in the same geographic location were collapsed into one clinic.

The study was approved by Stellenbosch University’s Health Research Ethics Committee and Boston University’s Institutional Review Board. Given the study’s retrospective nature, an informed consent waiver was granted by Stellenbosch University’s Health Research Ethics Committee and Boston University’s Institutional Review Board. All research was performed in accordance with relevant guidelines and regulations.

### Statistical and spatial analysis

We used descriptive statistics to examine the sample demographics across the different drug-resistant TB groups (RR-TB, pre-XDR-TB/XDR-TB, and XDR-TB). We compared the demographics of individuals with RR-TB only with those who had XDR-TB using chi-squared tests (for categorical variables) and t-tests (for continuous variables). For these comparisons, an “RR-TB only” group was used that excluded forms of pre-XDR and XDR-TB so that we were comparing independent groups. Then, we examined the distribution of the three TB groups (RR-TB, pre-XDR-TB/XDR-TB, and XDR-TB) at the district and subdistrict level. We defined “percentage XDR-TB” as the number of individuals with XDR-TB divided by the number of individuals with RR-TB; similarly, the “percentage pre-XDR-TB/XDR-TB” is the number of individuals with pre-XDR-TB or XDR-TB divided by the number of individuals with RR-TB. We estimated the percentage XDR-TB and percentage pre-XDR-TB/XDR-TB at the subdistrict-level and generated descriptive maps across the Western Cape province.

In addition, we estimated the percentage XDR-TB and the percentage pre-XDR-TB/XDR-TB at each clinic in Cape Town and used standard inverse distance weighting (IDW) heatmaps to visualize the percentages XDR-TB and pre-XDR/XDR-TB in the Cape Town metropole. We also created amikacin-resistant TB and ofloxacin-resistant TB IDW heatmaps. We generated IDW heatmaps of “count XDR-TB”, defined as the number of individuals with XDR-TB, and of “count pre-XDR-TB/XDR-TB”, defined as the number of individuals with pre-XDR-TB or XDR-TB. We also conducted the same analysis on only amikacin-resistant TB and on only ofloxacin-resistant TB.

IDW heatmaps assign greater influence to nearby points and less influence to further points, assuming that the number or percentage of individuals with XDR-TB by clinic reflects the XDR-TB distribution proximal to the clinic. IDW was only used in the Cape Town metropole, because outside Cape Town many clinics were spread far and several had low case counts, which would increase the bias of IDW and making it difficult to interpret the results. In addition, we conducted hotspot analysis using Getis-Ord Gi* in Cape Town to identify hotspots and coldspots for both of our outcomes of interest (percentage and count). We define hotspots as areas with significantly greater percentage or count than would be expected by chance. A coldspot was a spatial area with a significantly lower percentage or count than would be expected by chance. Statistical significance was reached when there is a higher than expected risk within the geographic area of interest compared to the average across the region.

### Sensitivity analysis

We assessed whether our findings were robust to differential testing for additional drug resistance and small clinic sizes. Bias could be introduced by small numbers of individuals with RR-TB per clinic or differential drug susceptibility testing practices, as these factors could produce the false findings of spatial heterogeneity. Clinics with only a few individuals with RR-TB could result in biased percentages of XDR-TB and pre-XDR/XDR-TB either upwards or downwards. Furthermore, clinics with a low percentage of individuals with RR-TB that received second-line DST, hereafter referred to as “second-line DST percentage”, would diagnose fewer individuals with XDR-TB or pre-XDR/XDR-TB, which would artificially bias the percentages downward. To assess potential bias due to small clinic sample size, we conducted a Pearson correlation to assess whether there was an association between the number of individuals with RR-TB and percentage XDR-TB and pre-XDR/XDR-TB at the clinic-level in Cape Town. To assess potential bias due to low percentage second-line DST, we ran a Pearson correlation to examine the association of second-line DST percentage with percentage XDR-TB and pre-XDR/XDR-TB in Western Cape subdistricts and in Cape Town clinics. If any source of potential bias was detected, additional sensitivity analyses were conducted, such as excluding individuals with RR-TB without second-line DST.

**Figure 1:**
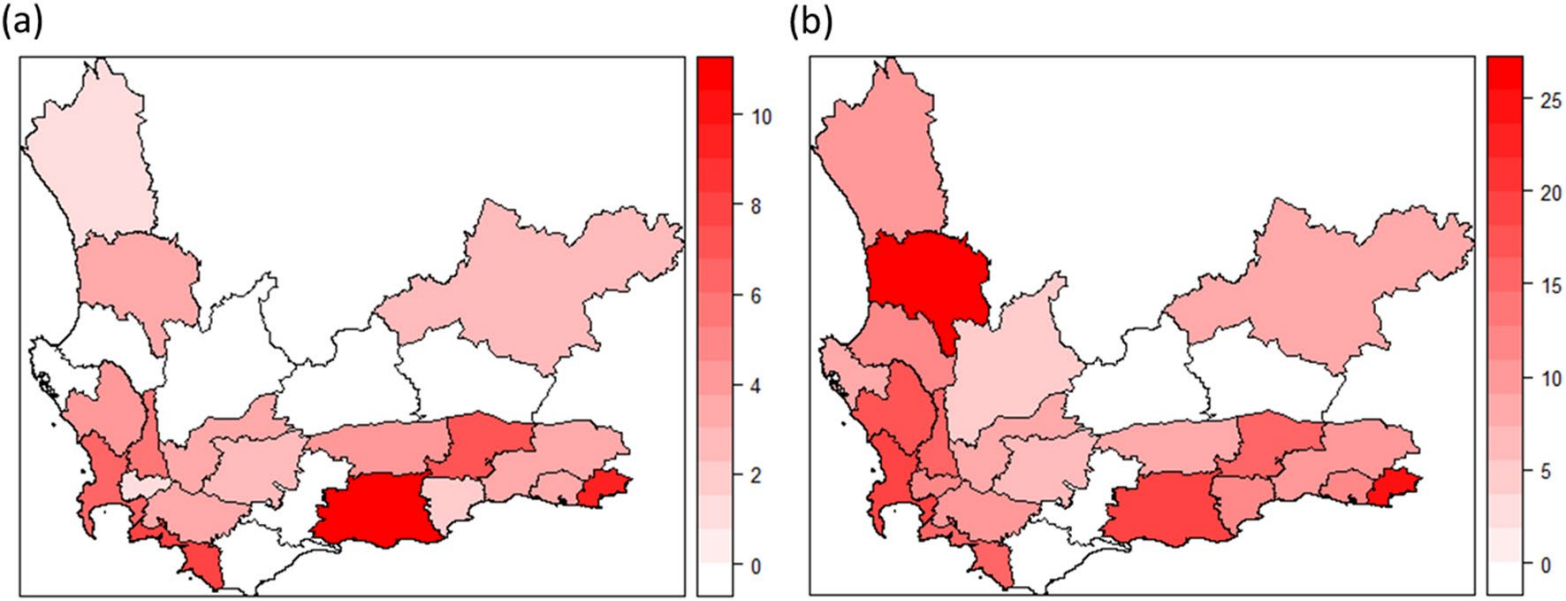
(**a**) Percentage XDR-TB in the Western Cape aggregated by subdistrict (**b**) pre-XDR/XDR-TB in the Western Cape aggregated by subdistrict. Figure created in R statistical software version 3.4.1^23^. *XDR-TB* extensively drug-resistant tuberculosis.

**Figure 2:**
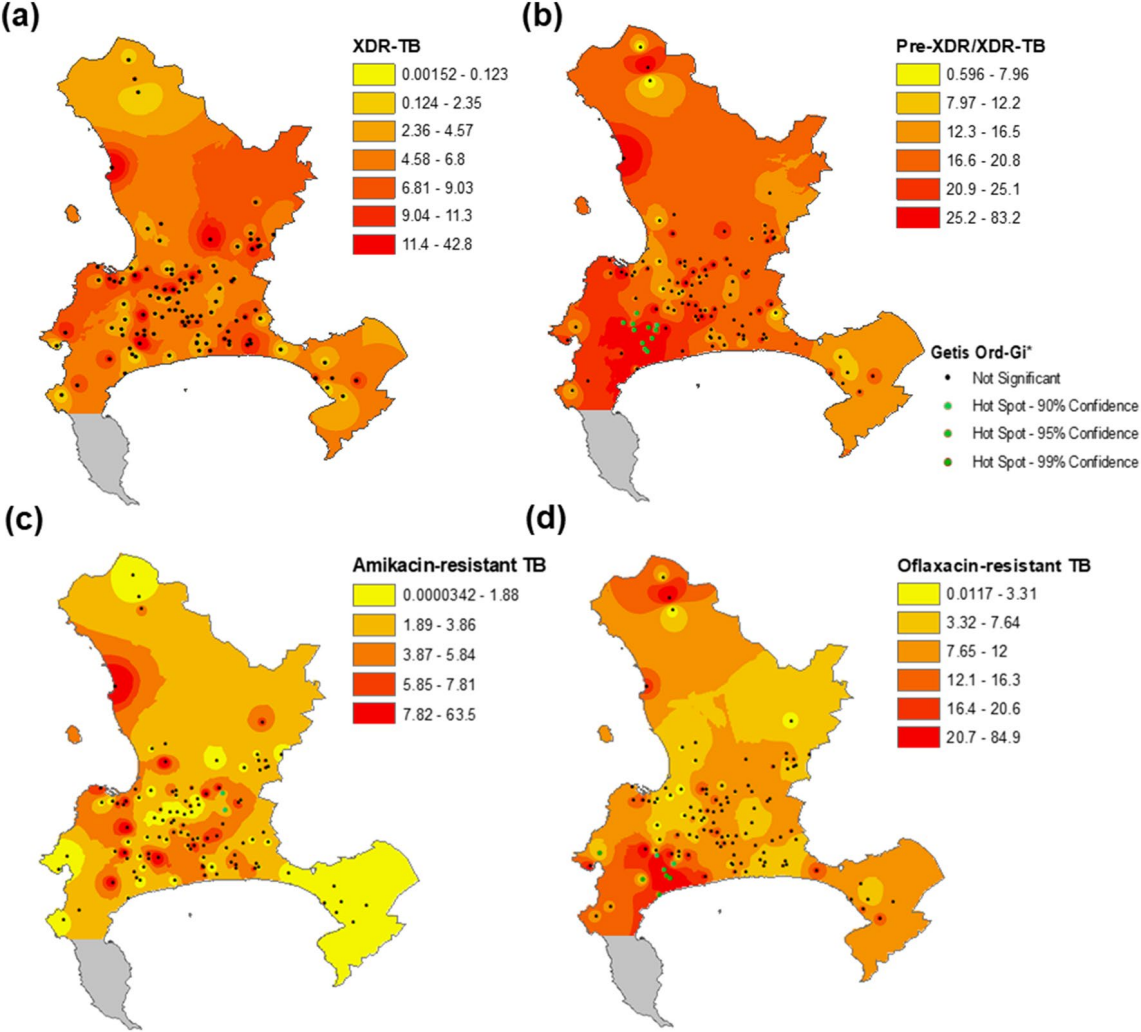
Inverse distance weighting map and Getis-Ord Gi* hotspot analysis of the percentage (**a**) XDR-TB, (**b**) pre-XDR/XDR-TB, (**c**) amikacin-resistant TB, and (**d**) ofloxacin-resistant TB in the city of Cape Town between 2012 and 2015. Figure created in ArcGIS version 10.8^24^. Colors are broken down by 1 standard deviation. Clinics are denoted as dots on the map, and each map is on a different scale. There are no clinics in Cape Point (grey).

**Figure 3:**
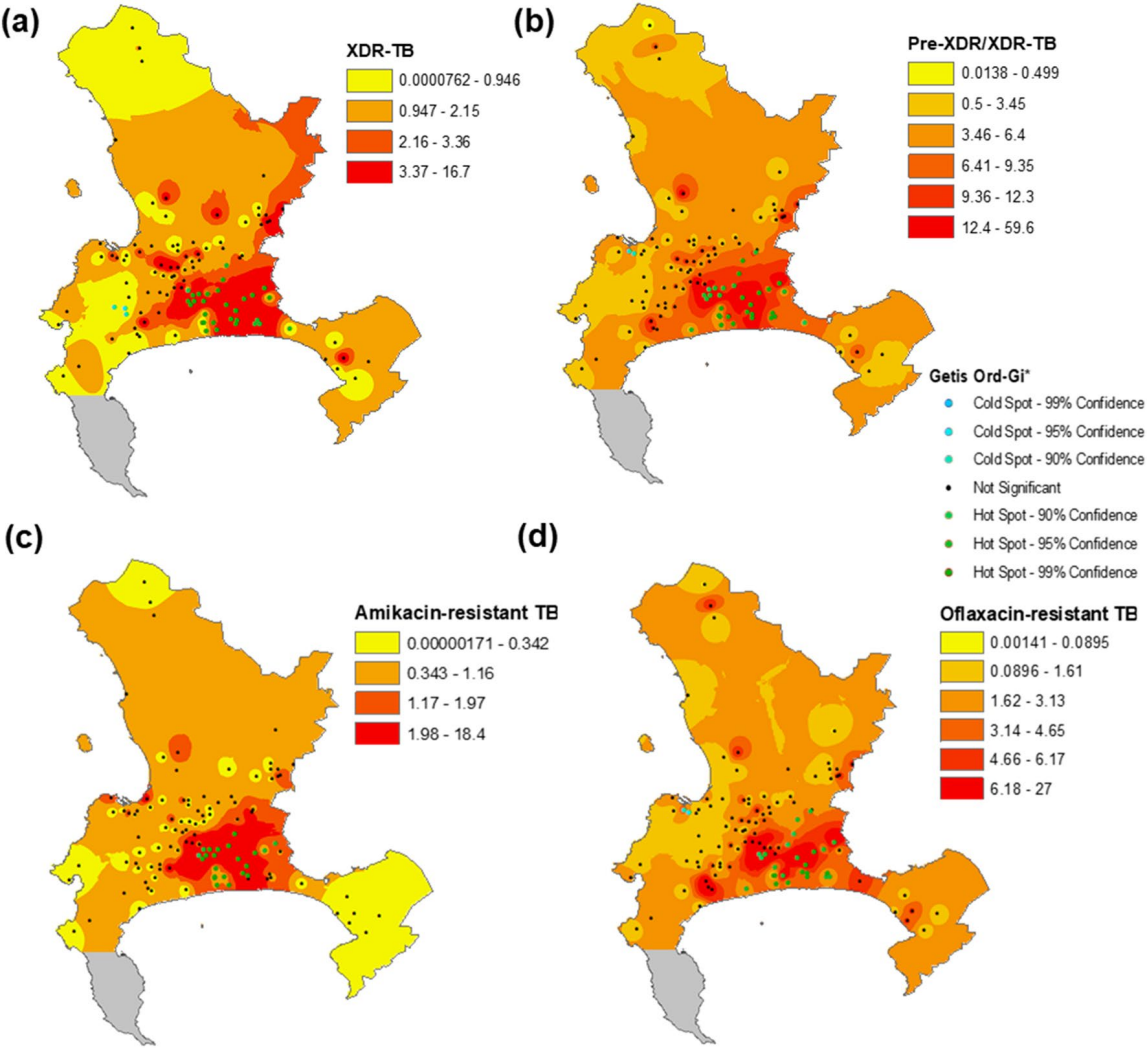
Inverse distance weighting map and Getis-Ord Gi* hotspot analysis of the counts of (**a**) XDR-TB, (**b**) pre-XDR/XDR-TB, (**c**) amikacin-resistant TB, and (**d**) ofloxacin-resistant TB in the city of Cape Town between 2012 and 2015. Figure created in ArcGIS version 10.8^24^. Colors are broken down by 1 standard deviation. Clinics are denoted as dots on the map, and each map is on a different scale. There are no clinics in Cape Point (grey).

All descriptive and sensitivity analyses, including descriptive mapping (Fig. 1), were conducted in R statistical software version 3.4.1^23^. The spatial IDW heatmap were created (Figs. 2, 3, and Supp. Fig. 2) and hotspot analyses were conducted in ArcGIS version 10.8^24^.

## Results

### Participants

Between January 1, 2012 and July 31, 2015, 430,969 people were evaluated for TB in the Western Cape province and recorded in NHLS, of whom 93,619 (21.7%) were diagnosed with TB. Among the individuals with TB, 6986 (7.4%) had laboratory-confirmed RR-TB by either DST, LPA, or Xpert. Of these, 2878 (41.2%) exclusively provided samples at clinics, and 3423 (49.0%) provided samples at clinics and other locations; 696 (10.0%) were not mappable to a clinic location and were excluded from the analysis. Thus, the final cohort included 6301 individuals with RR-TB (90.2% of 6986), associated with 280 clinics. Of the 6301 mappable individuals with RR-TB, 923 (15%) were not diagnosed with RR-TB at a clinic location, and thus we extracted the clinic visit closest in time to their RR-TB diagnosis. Supplementary Fig. 1 shows the flow diagram from initial specimens to the final RR-TB cohort.

### Descriptive analysis

Among the 6301 individuals with RR-TB mappable to a clinic, 701 (11.1%) and 340 (5.4%) had pre-XDR-TB and XDR-TB, respectively. Of the 701 with pre-XDR-TB, 515 (73.5%) had resistance to ofloxacin and 186 (26.5%) had resistance to amikacin. The percentage of RR-TB cases that had second-line DST reported was 82% (n = 5162), with 10 RR-TB cases only tested for only either ofloxacin or amikacin. (Supplementary Fig. 1). We included all RR-TB individuals in the main analysis, and conducted a sensitivity analysis removing RR-TB individuals with missing second-line DST. On average, individuals with XDR-TB were similar in age to those with RR-TB only, 33 and 34 years respectively; 43.8% and 41.7% of those with XDR-TB and RR-TB only respectively were female (Table 1). Individuals with XDR-TB more often had smear positive disease (79.4%) compared to those with RR-TB only (65.9%) (p < 0.0001).

**Table 1:** Baseline characteristics of individuals with RR-TB (mappable to clinic) in Western Cape, South Africa.

	All (n = 6301)		XDR-TB only (n = 340)		Pre-XDR/XDR-TB (n = 1041)		RR-TB only (n = 5260)	
	Median	95% CI	Median	95% CI	Median	95% CI	Median	Median
Age (years)	34	19–55	33*	26–41*	33*	25–42*	34	27–43

	n	%	n	%	n	%	n	%
**Gender**								
Male	3419	54.3	179	52.6	565	54.3	2854	54.3
Female	2627	41.7	149	43.8	434	41.7	2193	41.7
Unknown	255	4.0	12	3.5	42	4.0	213	4.0
**Smear status**								
Smear +	4293	68.1	270*	79.4*	827*	79.4*	3466	3466
**TB diagnostic test**								
Xpert	2130	33.8	119	35.0	340	32.7	1790	34.0
Culture	2422	38.4	131	38.5	392	37.7	2030	38.6
Smear	1700	27.0	89	26.2	304	29.2	1396	26.5
RT-PCR	49	0.8	1	0.3	5	0.5	44	0.8
**RR-TB diagnostic test**								
Xpert	2033	32.3	111	32.6	332	31.9	1701	32.3
LPA	4267	67.7	229	67.4	708	68.0	3559	67.7
DST	1	0.0	0	0.0	1	0.1	0	0.0
**Second-line DST**								
Yes	5162	81.9	340*	100*	1041*	31.9*	4121	78.3

*Significant p-value (XDR-TB group or pre-XDR/XDR group vs. RR-TB only) based on Wilcoxon t-test or Pearson chi-square test.

*LPA* line probe assay, *DST* drug susceptibility testing, *XDR-TB* extensively drug-resistant tuberculosis, *RR-TB* rifampicin resistant tuberculosis.

### Spatial heterogeneity in the number of individuals with XDR-TB and pre-XDR-TB/XDR-TB

Numbers of individuals with RR-TB, XDR-TB, and pre-XDR/XDR-TB were variably distributed across the Western Cape districts (Table 2). The highest number of individuals with XDR-TB were in the City of Cape Town, where 76.8% of individuals with XDR-TB in the Western Cape live. Furthermore, 73.2% and 64.4% of individuals with pre-XDR/XDR-TB and RR-TB were found in the City of Cape Town, indicating a higher XDR-TB and pre-XDR/XDR-TB individual concentration in Cape Town compared to RR-TB. We also found heterogeneity in the number of XDR-TB and pre-XDR-TB/XDR-TB individuals at the subdistrict level (see Supplementary Table 2).

**Table 2:** Number and percentage of individuals with RR-TB, XDR-TB, and pre-XDR/XDR-TB in the Western Cape districts.

District	XDR-TB only			Pre-XDR/XDR-TB			Total RR-TB	
	n	% of XDR-TB in Western Cape (%)	% of RR-TB in district	n	% of XDR-TB in Western Cape (%)	% of RR-TB in district (%)	n	% in Western Cape (%)
Cape Winelands	26	7.6	3.4	85	8.2	11.0	775	12.3
Central Karoo	1	0.3	1.2	5	0.5	6.0	84	1.3
City of Cape Town	261	76.8	6.4	762	73.2	18.8	4055	64.4
Eden	30	8.8	5.4	80	7.7	14.3	560	8.9
Overberg	9	2.6	3.8	23	2.2	9.7	237	3.8
West Coast	13	3.8	2.2	86	8.3	14.6	590	9.4
Total	340	100		1041	100		6301	100

*XDR-TB* extensively drug-resistant tuberculosis, *RR-TB* rifampicin resistant tuberculosis.

### Spatial heterogeneity in the percentage XDR-TB and percentage pre-XDR-TB/XDR-TB

There was considerable spatial heterogeneity in the distribution of the percentage XDR-TB and pre-XDR/XDR-TB across the Western Cape, both at the district and subdistrict-level. At the district-level, the percentage XDR-TB ranged from 1.2% to 6.4%, and the percentage pre-XDR-TB/XDR-TB ranged from 6.0 to 18.8%. The city of Cape Town had the greatest percentage XDR-TB (76.8%) and pre-XDR/XDR-TB (73.2%) relative to the other districts (0.3 to 8.8% for XDR-TB, and 0.5 to 8.3% for pre-XDR/XDR-TB) (Table 2). In Cape Town subdistricts, the percentage XDR-TB ranged from 0 to 10.5% (Fig. 1a), and the percentage pre-XDR/XDR-TB spanned 0–27.2% (Fig. 1b). Supplementary Table 1 provides a detailed breakdown of these percentages at both the subdistrict and district-levels.

### Spatial analysis in Cape Town

The Western Cape province included 406 clinics diagnosing TB, of which 280 clinics had at least one individual with RR-TB in the database^25^. Of these 280 healthcare clinics, 110 (39.3%) were in the city of Cape Town. In the city of Cape Town, the median percentage XDR-TB over all clinics was 4.9% (IQR 0–8.6%) (Fig. 2a), and the median percentage pre-XDR/XDR-TB was 17.5% (IQR 12.3–25.0%) (Fig. 2b). Figure 2 also illustrates the heterogenous distribution of amikacin-resistant TB (Fig. 2c) and ofloxacin-resistant TB (Fig. 2d) percentage among RR-TB within Cape Town clinics.

In the Getis-Ord-G* hotspot analysis of percentage XDR-TB and percentage pre-XDR/XDR-TB in Cape Town, no hot or coldspots of percentage XDR-TB were identified (Fig. 2a), while 11 hotspots of percentage pre-XDR/XDR-TB were found (Fig. 2b). There were two and eight hotspots of percentage amikacin-resistant and percentage ofloxacin-resistant TB (Fig. 2c,d), respectively. The percentage ofloxacin-resistant TB hotspots occur in similar areas to percentage pre-XDR/XDR-TB hotspots, while percentage amikacin-resistant hotspots occur in different areas.

The same analysis was conducted on count XDR-TB and count pre-XDR-TB/XDR-TB; 31 clinic hotspots and 3 coldspots of count XDR-TB were identified (Fig. 3a), and there were the same numbers of hot and coldspots of count pre-XDR/XDR-TB (Fig. 3b). For count amikacin-resistant TB, there were 22 hotspots, while there were 25 hotspots and 2 coldspots of count ofloxacin-resistant TB (Fig. 3c,d). There is substantial overlap between count amikacin-resistant and count ofloxacin-resistant TB hotspots, as well as with count XDR-TB and count pre-XDR/XDR-TB hotspots.

### Sensitivity analysis

#### Size of RR-TB in clinics and second-line DST percentages

The median number of individuals with RR-TB per clinic was 23.5 (IQR 9–49) over the study period. There was no association between number of individuals with RR-TB and percentage XDR-TB (p = 0.69) and percentage pre-XDR/XDR-TB (p = 0.38) at the clinic-level in Cape Town. Among subdistricts, the median percentage of RR-TB cases that had second-line DST was 82.1% (IQR 77.3–83.9%); in Cape Town clinics, the median was 82.6% (IQR 76.4–88.4%). There was no association between percentage of RR-TB with second-line DST and percentage XDR-TB (r = 0.21, p = 0.24) and pre-XDR/XDR-TB (r = 0.21, p = 0.24) at the subdistrict-level; however, they were positively associated at the clinic-level in Cape Town (r = 0.22, p = 0.019) (r = 0.37, p < 0.0001).

In post-hoc sensitivity analysis of the IDW heatmap and hotspot analysis in Cape Town, we excluded all individuals with RR-TB without second-line DST in order to assess the extent of bias in our results due to differential DST testing. Descriptive analysis, IDW and hotspot analysis using the percentage XDR-TB was reanalyzed using only RR-TB with second-line DST as the denominator. There were 5,162 individuals with RR-TB who had second-line DST. The median percentage XDR-TB at the clinic-level in Cape Town was 6.1% (IQR 0–10.5%), while the median percentage pre-XDR/XDR-TB was 21.8% (IQR 15.1–31.6%). The IDW map analysis of percentage XDR-TB, percentage pre-XDR/XDR-TB, count XDR-TB, and count pre-XDR/XDR-TB also showed a similar distribution as the previous analysis. There was substantial overlap in the hotspots of the Getis-Ord Gi* analysis for percentage XDR-TB, count XDR-TB, and count pre-XDR/XDR-TB (see Supplementary Fig. 2); however, there were no longer any hot spots in the percentage pre-XDR/XDR-TB in the sensitivity analysis (see Supplementary Fig. 2b), and there were no longer any cold spots detected in the count XDR-TB (see Supplementary Fig. 2c) and count pre-XDR/XDR-TB (see Supplementary Fig. 2d).

## Discussion

We found considerable spatial variation in the distribution of RR-TB and XDR- and pre-XDR/XDR-TB in the Western Cape Province, South Africa. The percentage RR-TB found to be second-line drug-resistant varied across subdistricts in the Western Cape and within clinics in the Cape Town Metropole. Hotspot analysis also confirmed statistically significant hot and cold spots for count XDR-TB and percentage and count pre-XDR/XDR-TB in Cape Town. Clinics with lower numbers of individuals with RR-TB and variation in second-line testing practices did not account for the spatial heterogeneity found in our study. The burden of second-line drug resistant TB (pre-XDR and XDR-TB) was also not uniformly distributed within the Western Cape, with concentration in the urban center of Cape Town. Previous research in KwaZulu-Natal has shown that there was a greater prevalence of XDR-TB in the eThekwini district, which is also a more urbanized area similar to the Cape Town metropole^26^.

Our findings demonstrate additional spatial clustering of second-line drug resistant TB cases even among individuals with RR-TB, and that RR-TB burden per se is not sufficient to predict areas with greater than expected XDR-TB burden. Our results also indicate that even on as small a scale as within a metropolitan region, XDR and pre-XDR/XDR cases have become more dominant differentially, whether driven by transmission or acquisition. Previous South African research has shown that around 70% of individuals with XDR-TB were infected with a genetically closely-related *Mtb* strain, rather than acquiring additional resistance during treatment for a less resistant strain^26^. The underlying reasons for increased transmission of XDR-TB specifically in some locations requires further research, but sequencing of *Mtb* samples collected from patients could help understand where and why transmission is occurring.

There was considerable overlap of hotspots between individuals with XDR-TB and pre-XDR/XDR-TB, which could indicate similar drivers of drug resistance emergence. There are several potential reasons for spatial clustering of second-line drug resistant TB. For example, social conditions that coalesce with disease transmission, such as homelessness, the HIV epidemic, high population density, and increased migration, and could all be associated with the clustering we observed here^19–23^. Access to appropriate support throughout treatment may also vary by location and could be driving spatial clusters of acquired XDR-TB. Moreover, previous research has found greater prevalence of TB and MDR-TB in urban settings in South Africa^13,15,18^. Future research needs to assess individual-level risk factors and area-level correlates of XDR-TB clusters.

Importantly, our finding that percentage ofloxacin-resistant TB hotspots reflect the spatial distribution of pre-XDR/XDR-TB hotspots suggest that fluoroquinolones more often may be the first drug to develop resistance in MDR/RR-TB. Hence, fluoroquinolone resistance may be a better signal for where pre-XDR-TB and evolving XDR-TB is occurring in the community. Our sample size of amikacin-resistant TB patients, though, may not have been large enough to identify a smaller number of hotspots where this resistance forms initially. Our results have important implications for the improvement of case detection; intensified case finding in XDR-TB hotspots would identify the various types of second-line drug resistant TB, due to the overlap of hotspots of count amikacin-resistant TB and count ofloxacin-resistant TB with count XDR-TB and count pre-XDR-TB hotspots.

The spatial heterogeneity of XDR-TB has previously been observed in other South African settings^5,13–15^. A novel aspect of our study is that we explicitly account for underlying MDR/RR-TB burden, teasing out that high XDR-TB burden varies across higher RR-TB burden areas. Previous research on the spatial distribution of XDR-TB in South Africa has been mostly done in KwaZulu-Natal, the province with very high XDR-TB burden and where XDR-TB originally was identified and defined^27^. The Western Cape province has much lower rates of people with HIV compared to KwaZulu-Natal^28^, which likely modifies the spatial distribution of TB. Individuals with HIV progress to disease more often and rapidly, meaning their disease reflects more recently circulating strains. Unfortunately, we do not have HIV status on individuals in our cohort so could not explore this further. Another strength of the study is that the NHLS data used is from a centrally collected laboratory database that processes nearly all TB tests^21^. We demonstrate the potential to leverage these routinely collected data for drug resistance burden surveillance and identify potential geographic hot spots for tracking and case finding efforts.

Our study has limitations. First, our current work does not incorporate associated genomic data, where linked clusters could be verified using pathogen genomics. Our approach is still valuable in that we see considerable spatial variation of various drug resistant TB that now support future molecular study. Second, in 2015 the South Africa TB treatment program introduced bedaquiline for treating individuals with RR-TB^29^. More recently, the definition of XDR-TB has changed to incorporate resistance to bedaquiline and linezolid, now recommended as first-line drugs for RR-TB treatment^30^. Tests for resistance to these two drugs were not performed on these specimens as these drugs were rarely given during our study period. Future research could use this approach to assess whether bedaquiline-resistance and resistance to other new drugs follows similar spatial patterns as that to ofloxacin and amikacin. Third, we omitted individuals with samples submitted only from non-clinic locations from our spatial analysis because non-clinic locations do not reflect an individual’s residence. With decentralization of RR/MDR-TB care in South Africa in 2011, which improved access and reduced costs of treatment^31,32^, we expect that the exclusion of non-clinic locations would not substantially affect our estimates since most MDR-TB care is now at the clinic-level. In addition, we assumed that clinic is a proxy for residence but it is possible that some patients traveled for care to a clinic less proximal to their residence (e.g. where they worked or had family living) and thus might not fully reflect residence. Lastly, a potential source of misclassification is from the person-matching algorithm, which approximately matched the specimens to the most likely individual. We would expect non-differential misclassification, which would not substantially change the distribution of drug resistant TB in our analysis.

The subnational variations in spatial distribution of percentage XDR-TB and percentage pre-XDR-TB/XDR-TB in the Western Cape province suggest that second-line drug resistance occurs unevenly, hitting specific areas harder than others. Our findings have implications for South African TB control, as preventing XDR-TB transmission could benefit from real-time monitoring of drug-resistant clusters for timely response measures. In 2017, the South African TB control program introduced Genotype MTBDR*sl* assay, and the roll-out of additional molecular SLD tests, such as Xpert MTB/XDR, would greatly improve domestic drug-resistant TB surveillance. The expansion of improved second-line drug resistance diagnostics creates a platform for continued effective monitoring and surveillance of pre-XDR and XDR-TB transmission.

## Supplementary Information

Supplementary Information.

## Data Availability

The datasets generated during and/or analysed during the current study are available in the Figshare repository, https://doi.org/10.6084/m9.figshare.17102636.v1.
