# Supplementary material for: Spatial heterogeneity of extensively drug resistant-tuberculosis in Western Cape Province, South Africa
Source: Sci Rep. 2022 Jun 27;12:10844. doi: 10.1038/s41598-022-14581-4 (PMC9237070; doi:10.1038/s41598-022-14581-4)
Supplement: Supplementary file 1 — Supplementary Information. [file 41598_2022_14581_MOESM1_ESM.docx]

**SUPPLEMENTARY MATERIAL**

**Spatial Heterogeneity of Extensively Drug Resistant-Tuberculosis in Western Cape Province, South Africa**

Karla Therese L. Sy^1^, Sarah V. Leavitt^2^, Margaretha de Vos^3^, Tania Dolby^4^, Jacob Bor^1,2^, C. Robert Horsburgh, Jr.^1,2,5,6^, Robin M. Warren^3^, Elizabeth M. Streicher^3^, Helen E. Jenkins^2*^, Karen R. Jacobson^6*^

**Table of contents**

**Supplementary Figure 1.** Flow diagram showing initial specimens from the Western Cape NHLS to final RR-TB cohort, January 2012 to July 2015

**Supplementary Table 1.** Distribution of XDR-TB, pre-XDR/XDR-TB, RR-TB with DST, and all RR-TB in the Western Cape districts and subdistricts

**Supplementary Figure 2.** Inverse distance weighting map and Getis-Ord Gi* hotspot analysis of (a) percentage XDR-TB (b) percentage pre-XDR/XDR-TB (c) count XDR-TB, and (d) count pre-XDR/XD-TB among cases with second-line drug susceptibility testing in the city of Cape Town between 2012 and 2015. Figure created in ArcGIS version 10.8 [24]

**Supplementary Figure 1.** Flow diagram showing initial specimens from the Western Cape NHLS to final RR-TB cohort, January 2012 to July 2015

**
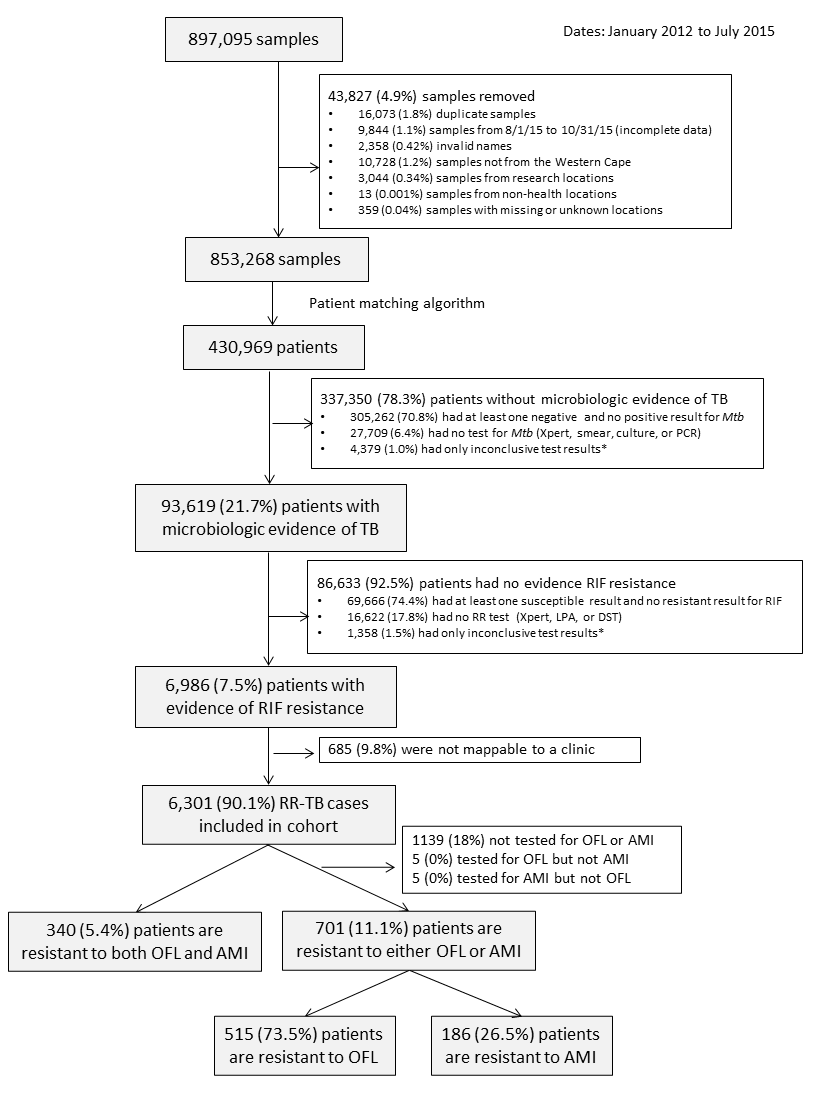
**

**Supplementary Table 1.** Distribution of XDR-TB, pre-XDR/XDR-TB, RR-TB with DST, and all RR-TB in the Western Cape districts and subdistricts

| **District and subdistrict** | **XDR-TB only** | | | **Pre-XDR/ XDR-TB** | | | **RR-TB with drug susceptibility testing (DST)** | | | **RR-TB** | |
| --- | --- | --- | --- | --- | --- | --- | --- | --- | --- | --- | --- |
|  | n | % of total XDR-TB | % of RR-TB in subdistrict | n | % of total pre-XDR/XDR-TB | % of RR-TB in subdistrict | n | % of total RR-TB tested | % of RR-TB in subdistrict | n | % of total RR-TB |
| **Cape Winelands** | **26** | **7.6** | **3.4** | **85** | **8.2** | **11.0** | **657** | **12.7** | **84.8** | **775** | **12.3** |
| Breede Valley | 7 | 2.1 | 3.5 | 18 | 1.7 | 9.0 | 167 | 3.2 | 83.9 | 199 | 3.2 |
| Drakenstein | 16 | 4.7 | 6.0 | 44 | 4.2 | 16.5 | 228 | 4.4 | 85.7 | 266 | 4.2 |
| Langeberg | 2 | 0.6 | 2.9 | 4 | 0.4 | 5.8 | 56 | 1.1 | 81.2 | 69 | 1.1 |
| Stellenbosch | 1 | 0.3 | 0.9 | 13 | 1.2 | 11.4 | 95 | 1.8 | 83.3 | 114 | 1.8 |
| Witzenberg | 0 | 0.0 | 0.0 | 6 | 0.6 | 4.7 | 111 | 2.2 | 87.4 | 127 | 2.0 |
|  |  |  |  |  |  |  |  |  |  |  |  |
| **Central Karoo** | **1** | **0.3** | **1.2** | **5** | **0.5** | **6.0** | **67** | **1.3** | **79.8** | **84** | **1.3** |
| Beaufort West | 1 | 0.3 | 1.4 | 5 | 0.5 | 7.2 | 57 | 1.1 | 82.6 | 69 | 1.1 |
| Laingsburg | 0 | 0.0 | 0.0 | 0 | 0.0 | 0.0 | 2 | 0.0 | 40.0 | 5 | 0.1 |
| Prince Albert | 0 | 0.0 | 0.0 | 0 | 0.0 | 0.0 | 8 | 0.2 | 80.0 | 10 | 0.2 |
|  |  |  |  |  |  |  |  |  |  |  |  |
| **City of Capetown** | **261** | **76.8** | **6.4** | **762** | **73.2** | **18.8** | **3333** | **64.6** | **82.2** | **4055** | **64.4** |
|  |  |  |  |  |  |  |  |  |  |  |  |
| **Eden** | **30** | **8.8** | **5.4** | **80** | **7.7** | **14.3** | **468** | **9.1** | **83.6** | **560** | **8.9** |
| Bitou | 8 | 2.4 | 10.5 | 19 | 1.8 | 25.0 | 62 | 1.2 | 81.6 | 76 | 1.2 |
| George | 8 | 2.4 | 4.1 | 20 | 1.9 | 10.3 | 165 | 3.2 | 85.1 | 194 | 3.1 |
| Hessequa | 4 | 1.2 | 10.5 | 8 | 0.8 | 21.1 | 28 | 0.5 | 73.7 | 38 | 0.6 |
| Kannaland | 1 | 0.3 | 4.2 | 2 | 0.2 | 8.3 | 19 | 0.4 | 79.2 | 24 | 0.4 |
| Knysna | 2 | 0.6 | 3.3 | 7 | 0.7 | 11.7 | 47 | 0.9 | 78.3 | 60 | 1.0 |
| Mossel Bay | 2 | 0.6 | 2.0 | 14 | 1.3 | 14.0 | 89 | 1.7 | 89.0 | 100 | 1.6 |
| Oudtshoorn | 5 | 1.5 | 7.4 | 10 | 1.0 | 14.7 | 58 | 1.1 | 85.3 | 68 | 1.1 |
|  |  |  |  |  |  |  |  |  |  |  |  |
| **Overberg** | **9** | **2.6** | **3.8** | **23** | **2.2** | **9.7** | **168** | **3.3** | **70.9** | **237** | **3.8** |
| Cape Agulhas | 0 | 0.0 | 0.0 | 0 | 0.0 | 0.0 | 17 | 0.3 | 77.3 | 22 | 0.3 |
| Overstrand | 5 | 1.5 | 7.8 | 10 | 1.0 | 15.6 | 44 | 0.9 | 68.8 | 64 | 1.0 |
| Swellendam | 0 | 0.0 | 0.0 | 0 | 0.0 | 0.0 | 23 | 0.4 | 82.1 | 28 | 0.4 |
| Theewaterskloof | 4 | 1.2 | 3.3 | 13 | 1.2 | 10.6 | 84 | 1.6 | 68.3 | 123 | 2.0 |
|  |  |  |  |  |  |  |  |  |  |  |  |
| **West Coast** | **13** | **3.8** | **2.2** | **86** | **8.3** | **14.6** | **469** | **9.1** | **79.5** | **590** | **9.4** |
| Bergrivier | 0 | 0.0 | 0.0 | 4 | 0.4 | 11.4 | 23 | 0.4 | 65.7 | 35 | 0.6 |
| Cederberg | 5 | 1.5 | 4.4 | 31 | 3.0 | 27.2 | 84 | 1.6 | 73.7 | 114 | 1.8 |
| Matzikama | 3 | 0.9 | 1.4 | 22 | 2.1 | 10.1 | 181 | 3.5 | 83.0 | 218 | 3.5 |
| Saldanha Bay | 0 | 0.0 | 0.0 | 8 | 0.8 | 8.3 | 69 | 1.3 | 71.9 | 96 | 1.5 |
| Swartland | 5 | 1.5 | 3.9 | 21 | 2.0 | 16.5 | 112 | 2.2 | 88.2 | 127 | 2.0 |
|  |  |  |  |  |  |  |  |  |  |  |  |
| **Total** | **340** | **100** | **5.4** | **1041** | **100** | **16.5** | **5162** | **100** | **81.9** | **6301** | **1.0** |

| **Supplementary Figure 2.** Inverse distance weighting map and Getis-Ord Gi* hotspot analysis of (a) percentage XDR-TB (b) percentage pre-XDR/XDR-TB (c) count XDR-TB, and (d) count pre-XDR/XD-TB among cases with second-line drug susceptibility testing in the city of Cape Town between 2012 and 2015. Figure created in ArcGIS version 10.8 [24] |  |  |  |  |  |  |  |  |  |  |  |  |  |
| --- | --- | --- | --- | --- | --- | --- | --- | --- | --- | --- | --- | --- | --- |


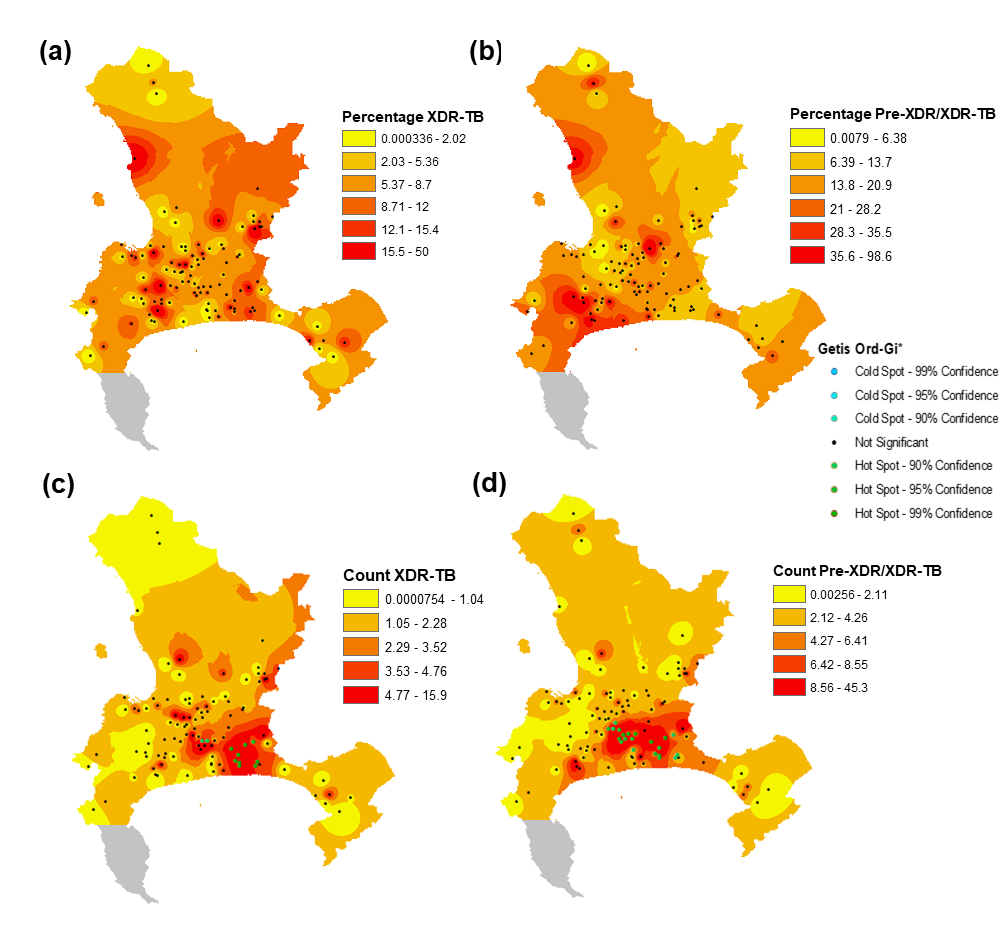


Colors are broken down by 1 standard deviation. Clinics are denoted as dots on the map, and each map is on a different scale. There are no clinics in Cape Point (grey).
